# Analysis of Risk Factors Associated With Central Lymph Node Metastasis in Papillary Thyroid Carcinoma With cT1N0 Stage

**DOI:** 10.3389/fendo.2022.880911

**Published:** 2022-06-03

**Authors:** Yin-zhu Zhao, Nian-an He, Xian-jun Ye, Fu Jin, Meng-xue Li, Xianxian Jiang

**Affiliations:** Department of Ultrasound Medicine, The First Affiliated Hospital of University of Science and Technology of China Anhui Provincial Hospital, Hefei, China

**Keywords:** papillary thyroid carcinoma, BRAF^V600E\^ mutation, central lymph node metastasis, risk factors, surgery

## Abstract

**Aim:**

Annual T1 stage papillary thyroid carcinoma (PTC) incidence rates continue to rise, yet the optimal treatment for this cancer type remains controversial. Central lymph node metastasis (CLNM) is a critical determinant in the context of treatment decision-making. While several prior studies have evaluated patients with clinica l T1a(cT1a) stage PTC, there have been fewer analyses of clinical T1b(cT1b) disease to date. The present study was thus formulated to explore predictors of CLNM in patients with cT1a and cT1b stage PTC.

**Methods:**

A retrospective analysis of data including clinicopathological characteristics and BRAF^V600E^ mutation status was conducted for 452 PTC patients undergoing surgical treatment. Logistic univariate and multivariate analyses were performed to identify risk factors associated with CLNM in particular patients’ characteristics and the accuracy of the established logistic regression models was evaluated using the R software platform.

**Results:**

Respective CLNM incidence rates in cT1a and cT1b disease were 39.39% and 67.21%. Factors associated with a higher risk of CLNM among PTC(cT1a) patients included male sex, young age, tumor size, contact with capsule, and multifocality as determined through comparisons of the area under the curve for logistic regression models. Whereas male sex and age were associated with CLNM risk in PTC(cT1b) patients in univariate and multivariate analyses, age was the only risk factor associated with CLNM incidence among women with PTC(cT1b).

**Conclusion:**

Predictors of CLNM differ between PTC patients with cT1a and cT1b stage disease, and a comprehensive assessment of these risk factors should thus be conducted when designing individualized treatment regimens for PTC patients.

## Introduction

Thyroid cancer remains an important cause of global morbidity, in large part owing to the rapid increase in the incidence of papillary thyroid cancer (PTC) and papillary thyroid microcarcinoma (PTMC) (1, 2), which is defined by PTC tumors ≤ 10 mm in diameter. At present, ultrasonography (US) is recommended as an initial auxiliary approach when seeking to differentiate between benign and malignant thyroid nodules. The combination of routine health assessments and US-guided fine-needle aspiration biopsy (FNAB) approaches have contributed to significant increases in rates of PTC detection and preoperative diagnosis (3, 4). At present, the AJCC and UICC TNM staging systems for differentiated thyroid carcinoma (DTC) classify T1 stage disease based upon an intrathyroidal tumor size ≤ 2 cm, with these tumors being further subdivided into T1a (≤ 1 cm) and T1b (> 1 cm but ≤ 2 cm) stage disease (5, 6). The incidence of clinical T1 (cT1) stage PTC as defined by pre-operative imaging studies and physical exam, has steadily increased in recent years (7). As PTC has an excellent prognosis in comparison to other malignancies, particularly for PTMC, a range of therapeutic approaches are recommended to patients with PTC(cT1N0) disease including active surveillance, radiofrequency ablation, and surgery.

However, certain factors are associated with poorer PTC patients prognosis, with central lymph node metastasis (CLNM) being an important risk factor that must be considered when evaluating patient risk (8, 9), given that such metastases have been tied to increases in rates of local recurrence and the risk of a second surgery. CLNM is a common finding that affects an estimated 13.4% - 64% of PTC patients (10–12). Owing to anatomical factors including the carotid pulse and the potential for small metastases not detectable *via* imaging examination to be present, the preoperative diagnosis of CLNM can be challenging. Prophylactic central lymph node dissection (pCLND) in the absence of clinical evidence of CLNM is associated with higher rates of surgery-related complications including recurrent laryngeal nerve injury and permanent hypoparathyroidism. As such, radiofrequency ablation or active surveillance are generally the management approaches of choice for low-risk PTC(T1) patients without any evidence of cervical lymph node involvement (13, 14). It is thus critical that risk factors related to CLNM incidence in patients with PTC be better defined in order to guide clinical decision-making. Several studies to date have assessed PTMC(T1a) patients and identified potential clinical and US features associated with CLNM risk, including male sex, younger age multifocality, and extrathyroidal extension, but these findings have not been consistent across studies and analyses of individuals with cT1b stage disease are currently lacking (15–17).

Several genetic mutations have been linked to the pathogenesis of PTC at the molecular level, and these genes can thus be examined to guide the diagnosis and prognostic evaluation of patients with PTC. The BRAF^V600E^ kinase mutation has been shown to be the most critical candidate biomarker in this oncogenic context owing to its ability to promote PTC onset and progression (18, 19). However, the association between BRAF^V600E^ mutation and CLNM prevalence remains controversial (19–21). BRAF^V600E^ mutation alone is thus not sufficient to accurately predict patient CLNM status, underscoring the need to define a panel of clinical risk factors associated with CLNM status in an effort to guide more accurate and effective patient treatment.

In the present retrospective analysis, we assessed the clinical features, preoperative ultrasonographic findings, BRAF^V600E^ mutational status, and postoperative clinicopathological features of cT1N0 stage PTC patients in an effort to identify predictors of CLNM. Together, these results provide a valuable foundation for efforts to more precisely and accurately plan treatments for PTC patients with cT1N0 stage disease.

## Materials and Methods

### Patient Demographics

For the present retrospective study, we analyzed data from PTC patients who had undergone thyroid lobectomy or total thyroidectomy with routine prophylactic central lymph node dissection (pCLND) between December 2020 and January 2022. The present study was approved by Review Board of our hospital, experienced thyroid surgeons performed all surgical procedures in order to ensure the integrity of the central lymph node dissection procedure.

In an effort to offer more reliable and valuable treatment options to patients with PTC, separate univariate and multivariate analyses were conducted for individuals with stage cT1a and stage cT1b disease. Individual patient groups were separated into individuals with and without CLNM based on postoperative clinicopathological findings.

Inclusion criteria for the present study were: (1) availability of complete preoperative and postoperative data; (2) postoperative pathological confirmation of the PTC diagnosis; (3) patients had undergone unilateral thyroidectomy or total thyroidectomy and central lymph node dissection; and (4) patients had undergone FNA and BRAF^V600E^ mutational status analyses. Patients were excluded from this study if they met the following criteria: (1) a history of neck irradiation, neck surgery, or radioactive iodide treatment; (2) an absence of histologic results pertaining to the thyroid and/or central lymph nodes; (3) pregnancy; (4) other head and neck tumors.

### Conventional and Color Doppler Ultrasonography

A sonographer with over 10 years of experience performed all imaging. Patients were directed to lie in the supine position with dorsal flexion of the head, and malignant thyroid node findings were identified *via* conventional US as per the TI-RADS criteria or according to the ATA guidelines (22, 23). Thyroid nodule size was assessed based on the maximum tumor diameter, with the presence or absence of the following features additionally being assessed (yes/no): aspect ratio>1, defined border, contact with the capsule, regular margin, microcalcification, and multifocality. After conventional grayscale ultrasonography, color Doppler ultrasound imaging was performed to detect the presence or absence of perinodular and intramodular vascular distributions.

### Fine Needle Aspiration, BRAF^V600E^ Detection, Surgical Methods

For FNA procedures, patients were directed to lie on their back with their neck hyperextended to ensure sufficient exposure of the puncture site. Very fine needles (23G) were then repeatedly inserted into the suspect node under ultrasonographic guidance to facilitate the acquisition of sufficient tissue. A portion of the collected tissue was used for cytological diagnosis, while the remainder was stored at 2-8°C. BRAF^V600E^ mutational status was assessed following DNA extraction *via* real-time fluorescent quantitative polymerase chain reaction.

Unilatearl thyroidectomy and isthmusectomy were performed when the tumor was located on a single lobe. Total thyroidectomy was performed when there were bilateral tumors, presence of extrathyroidal extension during intraoperative examination. Ipsilateral pCLND was performed when the tumor was unilateral, bilateral pCLND was performed in bilateral tumors. The boundary of pCLND was defined by the carotid arteries laterally, hyoid bone superiorly and suprasternal notch inferiorly, including pre- and paratracheal nodes, relaryngeal lymph nodes, perithyroidal nodes, and lymph nodes along recurrent laryngeal nerves. Parathyroid gland and recurrent laryngeal nerve were protected during procedure.

### Statistical Analysis

SPSS 26 (IBM Corporation, NY, USA) was used for statistical analyses. Categorical data were reported as frequencies and compared using chi-squared tests or Fisher’s exact test, while quantitative data are given as means ± standard deviation (SD) and compared *via* independent sample t-tests or Mann-Whitney U-tests when normally and non-normally distributed, respectively.

Univariate and multivariate logistic regression analyses were used to compare clinical characteristics, thyroid US features, and BRAF^V600E^ mutation status, with P < 0.05 as the threshold of significance. Those risk factors related to CLNM in univariate and multivariate analyses were employed to establish logistic regression models, with area under the curve (AUC) values being compared to confirm model accuracy. All such analyses were performed using the pROC package for the R software platform (R Foundation; v 4.1.2).

## Results

### Patient Characteristics

In total, this retrospective analysis incorporated data from 452 patients (113 male, 339 female), all of whom were preoperatively confirmed to have PTC and underwent US-guided FNA. The median age of these patients was 42.64 ± 11.13 years, and all had cT1N0M0 stage disease, including 330 patients (73.01%) with stage cT1a disease and 122 patients (26.99%) with stage cT1b disease. Overall, 46.9%(212/452) of patients had occult central lymph node metastases, with a significant difference in CLNM rates between males (61.95%; 70/113) and females (41.89%; 142/339) (P<0.01). The overall BRAF**^V600E^** mutation rate in these patients was 88.27% (399/452), with no significant differences in this rate when comparing males (86.73%; 98/113) and females (88.79%; 301/339) (P=0.555). The rate of Bethesda III, V, VI in cT1a stage was 8.48% (28/330), 23.64%(78/330), 67.88%(224/330), respectively, and 2.46%(3/122), 12.30%(15/122), 85.24%(104/122) in cT1b stage, respectively. No patients exhibited temporary hematoma or other complications following FNA, transient hypoparathyroidism occured in bilateral CLND and the incidence was 31.25% (25/80), serum parathyroid hormone returned to normal at post-operation one week or 2 months. There was no permanent hypoparathyroidism and recurrent laryngeal nerve injury after operation. There were no significant differences in maximum measured dimension between US results (9.09 ± 4.32 mm) and pathological specimens (9.52 ± 5.76 mm)(p=0.246). There were also no significant differences in disease duration, which was defined as the time that from ultrasound detection of nodules to surgery, when comparing patients with and without CLNM in the cT1a and cT1b groups.

### Correlations Between Clinicopathological Parameters, Ultrasonographic Features, and CLNM in PTC (cT1a)

The association between PTC(cT1a) patient clinicopathological findings, US features, and CLNM status was next assessed. The overall CLNM incidence in the PTC(cT1a) patient population was 39.39% (130/330), with respective incidence rates of 54.76% (46/84) and 34.15% (84/246) in male and female patients. The median number of central neck lymph nodes (CNLN) harvested was 4.5(2-8), the rate of CLNM number>5 was 5.38%(7/130) in cT1a stage patients (**Table 1**). Factors significantly associated with CLNM status included male sex (P < 0.001), age (P < 0.001), tumor size (P < 0.001), contact with capsule (P < 0.001), regular margin (P=0.046), microcalcification (P=0.028), and multifocality (P < 0.001) (**Table 2**). Univariate analyses indicated CLNM to be significantly associated with risk factors including sex (P=0.001), age (P < 0.001), tumor size (P < 0.001), contact with capsule (P < 0.001), microcalcification (P < 0.001), and multifocality (P < 0.001). For the multivariate analyses, six variables statistically significant in the univariate analyses were included in the logistic regression model. In our model, multivariate analyses further confirming sex (P=0.003, 95%CI 1.331-4.198), age (P < 0.001, 95%CI 1.027-1.077), tumor size (P < 0.001, 95%CI 0.667-0.879), contact with capsule (P=0.021, 95%CI 1.110-3.679), and multifocality (P=0.015, 95%CI 1.155-3.906) to be independent predictors for CLNM (**Table 3**).

**Table 1:** Pathologic characteristics of central lymph node.

	T1a	T1b
Number of harvested LN (median, IQR )	4.5 (2-8)	6.0 (3-10)
Number of patients with metastatic LN (n)	130	82
≤2	91 (70.00%)	41 (50.00%)
3-5	32 (24.62%)	23 (28.05%)
>5	7 (5.38%)	18 (21.95%)

LN, lymph nodes; IQR, interquartile range.

**Table 2:** Basic clinicopathologic characteristics and US-FNA features PTC (cT1aN0).

Clinicopathologic features	CLNM NO. (%)	Non-CLNM NO. (%)	P-value
Total	130	200	–
Sex			
Male Female	46 (35.38) 84 (64.62)	38 (19.00) 162 (81.00)	**< 0.001**
Age (years, mean± SD)	39.48±9.831	44.95±11.019	**<0.001**
Disease duration			
(months, mean)	7.96	8.55	0.730
Tumor size			
(mm, mean± SD)	7.505±2.046	6.508±1.747	**<0.001**
Regular margin			
Y N	9 (6.92) 121 (93.08)	28 (14.00) 172 (86.00)	**0.046**
Aspect ratio>1			
Y N	62 (47.69) 68 (52.31)	117 (58.50) 83 (41.50)	0.054
Defined border			
Y N	30 (23.08) 100 (76.92)	55 (27.50) 145 (72.50)	0.369
Contact with capsule			
Y N	59 (45.38) 71 (54.62)	43 (21.50) 157 (78.50)	**<0.001**
Perinodular vascular			
Y N	27 (20.77) 103 (79.23)	31 (15.50) 169 (84.50)	0.219
Intranodular vascular			
Y N	73 (56.15) 57 (43.85)	118 (59.00) 82 (41.00)	0.609
Microcalcification			
Y N	97 (74.62) 33 (25.38)	126 (63.00) 74 (37.00)	**0.028**
Multifocality			
Y N	53 (40.77) 77 (59.23)	40 (20.00) 160 (80.00)	**<0.001**
BRAF^V600E^ mutation			
Y N	117 (90.00) 13 (10.00)	177 (88.50) 23 (11.50)	0.669
Bethesda classification			
III V VI	10 (7.69) 28 (21.54) 92 (70.77)	18 (9.00) 50 (25.00) 132 (66.00)	0.675
Hashimoto's thyroiditis			
Y N	34 (26.15) 96 (73.85)	54 (27.00) 146 (73.00)	0.865
Nodular goiter			
Y N	12 (9.23) 118 (90.77)	19 (9.50) 181 (90.50)	0.935

PTC, papillary thyroid carcinoma; US-FNA, ultrasound-guided fine-needle aspiration; CLNM, central lymph node metastasis; Y, yes; N, no.

Bold values, p<0.05.

**Table 3:** Univariate and multivariate analyses to compare the high-risk factors of CLNM in PTC (cT1aN0).

Clinicopathologic features	Univariate			Multivariate*		
	OR	95%CI	P	OR	95%CI	P
Sex	2.335	1.410-3.865	**0.001**	2.364	1.331-4.198	0.003
Age	1.050	1.027-1.073	**<0.001**	1.052	1.027-1.077	<0.001
Disease duration	1.003	0.988-1.018	0.730			
Tumor size	0.755	0.667-0.856	**<0.001**	0.766	0.667-0.879	<0.001
Shape	0.457	0.208-1.003	0.051			
Aspect ratio>1	0.647	0.415-1.009	0.55			
Margin	0.791	0.474-1.321	0.370			
Contact with capsule	3.034	1.872-4.917	**<0.001**	2.021	1.110-3.679	0.021
Perinodular vascular	1.429	0.807-2.530	0.221			
Intranodular vascular	0.890	0.569-1.391	0.609			
Microcalcification	1.726	1.059-2.813	**0.028**	1.013	0.580-1.770	0.963
Multifocality	2.753	1.683-4.505	**<0.001**	2.124	1.155-3.906	0.015
BRAFV600E mutation	0.855	0.417-1.755	0.670			
Hashimoto's thyroiditis	0.935	0.454-2.069	0.969			
Nodular goiter	0.963	0.451-2.058	0.923			

PTC, papillary thyroid carcinoma; CLNM, central lymph node metastasis; OR, odds ratio; CI, confidence interval.

*Variables that reached p < 0.05 in univariate analyses were included. Bold values, p<0.05.

**Figure 1:**
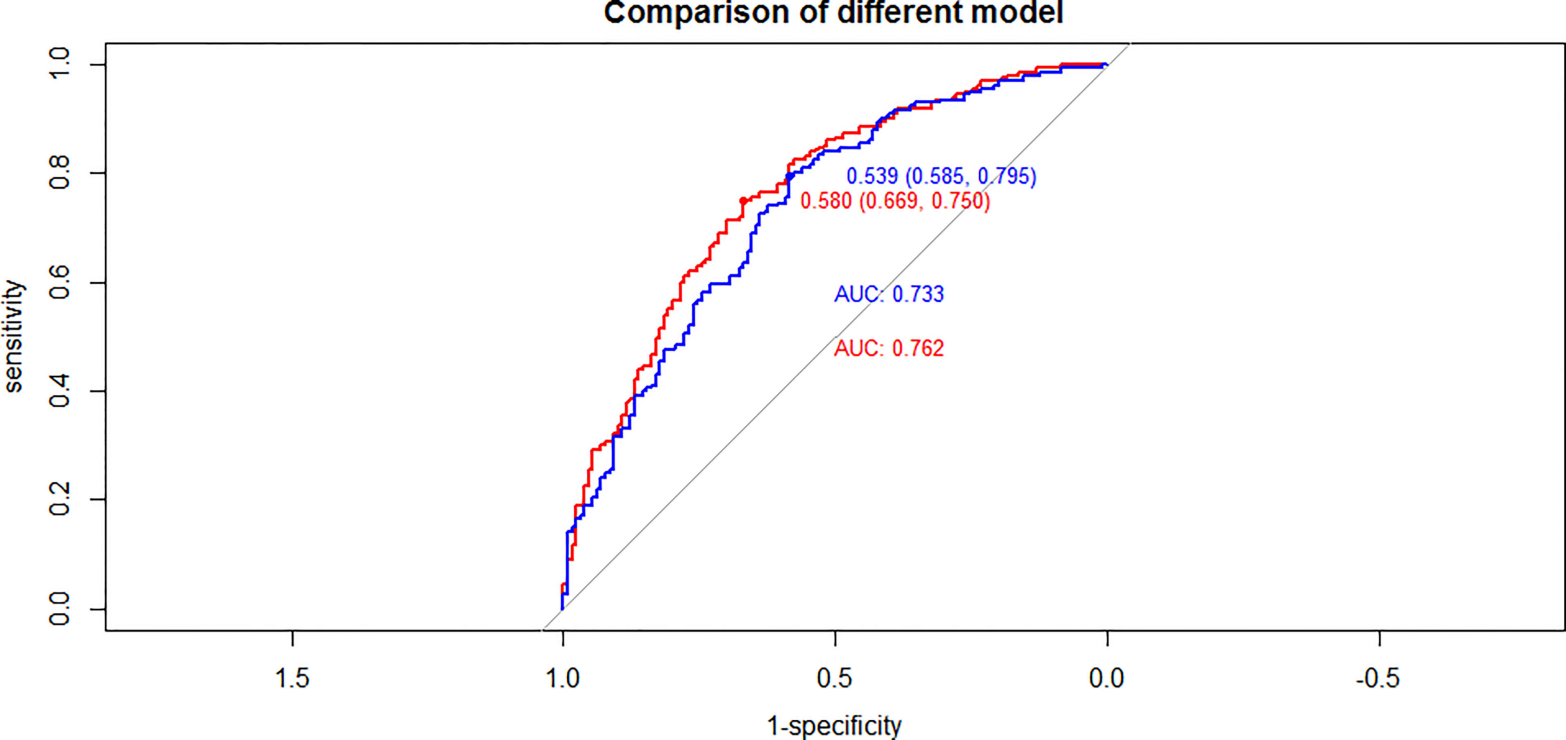
Compared of ROC curves between univariate and multivariate analyses results in PTC(cT1a) patients (red line: multivariate analyses results, blue line: univariate analyses results).

To further confirm the accuracy of these risk factors, we next established two regression models based upon the respective results of univariate and multivariate analyses and we then compared AUC values for these two models (**Figure 1**). The results of this comparison suggested the regression model established based upon multivariate analyses to be more accurate.

Given that both age and tumor size were identified as risk factors associated with CLNM incidence in PTC(cT1a) patients, efforts to more accurately define tumor size and age cut-off values are warranted to better predict CLNM. ROC curve analyses conducted based upon the above analyses revealed the optimal cut-off values for age and maximum tumor to be 44.5 years and 7.25 mm, respectively.

BRAF^V600E^ was positive in 294(89.09%) patients in the present study cohort, of whom 117 (90.00%) patients with CLNM and 177 (88.50%) patients without CLNM were positive for this BRAF**^V600E^** mutation. As such, while this BRAF^V600E^ mutation was common among PTC(cT1a) patients, it was not significantly related to CLNM incidence. Univariate analyses further revealed a significant association between Hashimoto’s thyroiditis (HT) and BRAF**^V600E^** mutation status.

### Correlations Between Clinicopathological Parameters, Ultrasonographic Features, and CLNM in PTC (cT1b)

Next, the risk factors associated with CLNM among PTC(cT1b) patients were identified. Overall CLNM incidence in this patient population was 67.21% (82/122), with respective 82.76% (24/29) and 62.37% (58/93) incidence rates in male and female patients, respectively. The median number of CNLN dissected was 6.0(3-10) and the rate of CLNM number> 5 was 21.95% (18/82) in cT1b stage patients (**Table 1**). Accordingly, male sex (P=0.041) was associated with CLNM incidence, as was age (P=0.022), wheras tumor size reached borderline statistical significance (p=0.057) (**Table 4**). In univariate analysis, male sex, and age were significantly associated with CLNM. For the multivariate analyses, variables (sex and age) were included in the logistic regression model, multivariate analyses further confirming sex(p=0.042, 95%CI 1.041-8.848), age(p=0.023, 95%CI 1.006-1.077) to be risk factors associated with CLNM incidence (**Table 5**).

**Table 4:** Basic clinicopathologic characteristics and US-FNA features PTC (cT1bN0).

Clinicopathologic features	CLNM (%)	Non-CLNM (%)	P-value
Total	82	40	–
Sex			
Male Female	24 (29.27) 58 (70.73)	5 (12.50) 35 (87.50)	**0.041**
Age (years, mean)	40.52±11.804	45.73±11.177	**0.022**
Disease duration			
(months, mean± SD)	9.49±15.576	12.16±23.214	0.454
Tumor size			
(mm, mean± SD)	15.396±3.186	14.170±3.539	0.057
Regular margin			
Y N	10 (12.20) 72 (87.80)	4 (10.00) 36 (90.00)	0.721
Aspect ratio>1			
Y N	25 (30.49) 57 (69.51)	15 (37.50) 25 (62.50)	0.439
Defined border			
Y N	18 (21.95) 64 (78.05)	10 (25.00) 30 (75.00)	0.707
Contact with capsule			
Y N	49 (59.76) 33 (40.24)	22 (55.00) 18 (45.00)	0.617
Perinodular vascular			
Y N	18 (21.95) 64 (78.05)	7 (17.50) 33 (82.50)	0.567
Intranodular vascular			
Y N	70 (85.37) 12 (14.63)	34 (85.00) 6 (15.00)	0.957
Microcalcification			
Y N	71 (86.59) 11 (13.41)	33 (82.50) 7 (17.50)	0.550
Multifocality			
Y N	20 (24.39) 62 (75.61)	9 (22.50) 31 (77.50)	0.818
BRAF^V600E^ mutation			
Y	68 (82.93)	37 (92.50)	0.152
N	14 (17.07)	3 (7.50)	
Bethesda classification			
III V VI	1 (1.22) 9 (10.98) 72 (87.80)	2 (5.00) 6 (15.00) 32 (80.00)	0.331
Hashimoto's thyroiditis			
Y N	29 (35.37) 53 (64.63)	15 (37.50) 25 (62.50)	0.818
Nodular goiter			
Y N	4 (4.88) 78 (95.12)	2 (5.00) 38 (95.00)	0.977

PTC, papillary thyroid carcinoma; US-FNA, ultrasound-guided fine-needle aspiration; CLNM, central lymph node metastasis; Y, yes; N, no.

Bold values, p<0.05.

**Table 5:** Univariate and multivariate analyses to compare the high-risk factors of CLNM in PTC (cT1bN0).

Clinicopathologic features	Univariate			Multivariate*		
	OR	95%CI	P	OR	95%CI	P
Sex	2.897	1.013-8.285	**0.047**	3.034	1.041-8.848	0.042
Age (years, mean)	1.039	1.005-1.074	**0.025**	1.040	1.006-1.077	0.023
Disease duration						
(months, mean± SD)	1.008	0.988-1.028	0.456			
Tumor size	0.892	0.791-1.004	0.059			
Shape	1.250	0.367-4.262	0.721			
Aspect ratio>1	0.731	0.330-1.618	0.439			
Margin	0.844	0.348-2.047	0.707			
Contact with capsule	1.215	0.566-2.607	0.617			
Perinodular vascular	1.326	0.503-3.494	0.568			
Intranodular vascular	1.029	0.356-2.978	0.957			
Microcalcification	1.369	0.487-3.849	0.551			
Multifocality	1.111	0.453-2.725	0.818			
BRAF^V600E^ mutation	2.539	0.685-9.408	0.163			

PTC, papillary thyroid carcinoma; CLNM, central lymph node metastasis; OR, odds ratio; CI, confidence interval.

*Variables that reached p < 0.05 in univariate analyses were included. Bold values, p<0.05.

Given the observed differences between males and females with respect to CLNM rates and the higher rates of CLNM among males in this patient population, we next conducted separate analyses of risk factors associated with CLNM in male and female PTC(cT1b) patients. This approach revealed that age was the only such risk factor identified among female patients, while no risk factors were identified for males in univariate analyses. Given the association between age and CLNM status, an ROC curve analysis was performed revealing the optimal age cut-off value for predicting CLNM status in female patients to be 44.5 years old.

BRAF^V600E^ mutation was positive in 105 (86.07%) of the analyzed cT1b PTC patients. No significant differences in BRAF^V600E^ mutation positivity were observed when comparing CLNM (82.93%) and non-CLNM (92.50%) patients (P=0.152), suggesting that while this mutation is common among individuals with PTC (cT1b), it is not specifically related to CLNM status in this patient population. Univariate analyses revealed a significant association between BRAF^V600E^ mutation and aspect ratio > 1 (P=0.047), contact with capsule (P=0.039), and HT (P=0.008). Multivariate analyses further confirmed BRAF^V600E^ mutation to be significantly associated with aspect ratio > 1 (P=0.045, 95%CI 0.042-0.962) and HT (P=0.025, 95%CI 1.179-11.254), whereas it was not significantly associated with contact with capsule (P=0.081, 95%CI 0.117-1.133).

## Discussion

The cervical lymph nodes are the most common site of metastasis in patients with PTC and PTMC (8), with CLNM being associated with an elevated chance of recurrent PTC and lower rates of conservative treatment for individuals diagnosed with PTMC (10, 11). In the present analysis, we observed inconsistencies in CLNM incidence between PTC patients with T1a and T1b disease (39.39% vs. 67.21%, respectively), in line with prior reports (9, 24). We also found the clinicopathological and ultrasonographic risk factors associated with CLNM in these two disease stage subgroups to differ significantly. In PTC(T1a) patients, male sex, age, tumor size, contact with capsule, and multifocality were identified as independent predictors of CLNM, whereas only age and male sex were independent predictors of CLNM among PTC(cT1b) patients. Incidence of CLNM>5 was low in cT1a stage, however, it was significantly higher in cT1b stage. Hence, we recommend that pCLND is not routinely done in cT1a stage. Together, these data have the potential to provide a valuable reference for efforts aimed at formulating more accurate surgical or non-surgical treatments for PTC patients with cT1 stage disease, thereby improving patient quality of life.

With respect to clinical characteristics, age and sex are risk factors associated with CLNM in PTC patients. Liu et al. proposed that males < 40 years of age were at an elevated risk of CLNM (25), whereas Tao et al. found being < 38 years old was associated with CLNM risk (8). Zhou et al. suggested that being male and under 30 years of age may be a more reliable cut-off when predicting the odds of CLNM in a Chinese patient population (16). However, these previous studies included patients with a range of tumor stages and failed to assess the association between age and tumor size within a given stage of diseases. Our present results suggested that an age of < 44.5 years and < 37.7 years for individuals with cT1a and cT1b disease, respectively, was associated with an increased risk of CLNM. When we conducted additional analyses of age among females with cT1b disease, being < 44.5 years was again confirmed to be associated with CLNM incidence among female patients, whereas age was not associated with CLNM risk among male patients with cT1b disease, probably due to high rate of CLNM. As such, further in-depth multi-center analyses of the association between age, sex, and CLNM are warranted, particularly for individuals with PTC(cT1b). A majority of these patients were asymptomatic with disease initially being detected *via* US examination, and as a result, no association between disease duration and CLNM was detected.

Tumor size has previously been linked to CLNM status, with larger tumors generally being more aggressive (21, 24, 25). In the present study, we compared differences between tumor sizes as measured *via* US and the actual sizes of pathological samples following surgical resection, revealing no differences between these sizes and thus confirming that US findings can be used when conducting predictive analyses. We found that tumors > 7.25 mm in size were associated with a high risk of CLNM among PTC(cT1a) patients, in line with prior research. Other US features have also been found to be associated with CLNM. For example, one meta-analysis found extrathyroidal extension to be associated with an elevated risk of CLNM among PTMC patients (12), while Wang et al. additionally conducted an analysis of 1204 patients with PTMC and found sonographic evidence of thyroid nodule microcalcification to be an independent predictor of CLNM (26). Tao et al. additionally identified a solid composition to be an independent factor of CLNM (8). While multivariate analyses in the present study revealed contact with capsule and multifocality to be independent predictors of CLNM among PTC(cT1a), no analyzed US features were related to CLNM risk in our PTC(cT1b) patient cohort. In light of these results, tumor size seems to offer greater value as a predictor of CLNM. Multifocality is commonly observed in PTC and is correlated with both CLNM and disease recurrence (27, 28), with overall reported tumor multifocality rates of 27% in a previous report (16), and 27.21% in our study, with our data confirming a correlation between such multifocality and CLNM for patients with cT1a stage disease.

Some reports have found BRAF^V600E^ mutation status to be related to CLNM incidence, whereas other studies have failed to replicate this finding. Here, we found BRAF^V600E^ mutation positivity to be highly prevalent among patients with both cT1a and cT1b stage disease (89.09% vs. 86.07%, respectively), with no significant difference is such positivity when comparing CLNM and non-CLNM patients in either stage subgroup, which indicated that BRAF^V600E^ mutation by itself did not contribute to the presence of CLNM in the absence of other clinico-pathologic factors.

BRAF^V600E^ mutation positivity was also found to be more common among PTMC patients without HT as compared to patients with comorbid HT in this study, in line with prior clinical evidence (16, 29).

The association between PTC and HT is poorly defined, as is the association between HT and CLNM incidence. In some reports, comorbid HT and PTC have been found to be associated with a lower risk of nodal metastasis as compared to that observed for patients not affected by HT (30, 31), whereas other studies have failed to detect an association between HT and CLNM (10, 32). In the present analysis, we were unable to detect any significant association between HT and CLNM in PTC patients with either cT1a or cT1b stage. Additional molecular mechanistic studies may thus be required to more thoroughly examine the interplay between PTC, HT, and CLNM.

There are multiple limitations to the present analysis. For one, this was a retrospective study and surgery is not recommended for the majority of such patients with tumors smaller than 5 mm in clinical practice, thus resulting in the maldistribution of PTMC patients, which is concentrated tumor diameter above 5mm. Second, this was a single-center study, and the number of enrolled patients with cT1b stage disease was limited. Patients in cT1b stage are more prone to capsule invasion and high rate of CLNM number >5, further large-scale multi-center studies will be critical as a means of more accurately defining risk factors associated with CLNM in this patient population. Moreover, information about the size of the largest metastatic deposit was not described, we will recommend that pathologists provide the size of positive lymph nodes in order to provide more accurate treatment for PTC patients in future study. Third, correlation analyses examining the associations between BRAF^V600E^ mutation status, progressive clinicopathological findings, and US features were limited by the numbers of patients with advanced-stage disease. Finally, because data on our cohort was collected fairly recently, we do not have enough followup time to determine the impact of pCLND or presence of CLNM on the overall prognosis of these PTC patients, particularly of those with cT1b disease. A prospective study with a longer duration of followup or a randomized control trial of pCLND vs no-pCLND may answer the true prognostic significance of occult CLNM in patients with cT1 disease.

## Conclusion

In conclusion, the results of this study highlight significant differences between PTC patients with cT1a and cT1b disease. Among PTC(cT1a) patients, independent predictors of CLNM included male sex, age, large tumor size (> 7.25 mm), contact with capsule, and multifocality, while among PTC(cT1b) patients these independent predictors included male sex and age. A range of analyses including ultrasonography, assessments of patient clinical characteristics, and US-FNAB should be performed prior to the establishment of a definitive patient treatment plan. As not all PTC(cT1N0) lesions are alike, the formulation of appropriate individualized therapeutic regimens is dependent on comprehensive analyses of all of these pertinent risk factors.

## Data Availability Statement

The raw data supporting the conclusions of this article will be made available by the authors, without undue reservation.

## Ethics Statement

The studies involving human participants were reviewed and approved by The Ethics Committee of The First Affiliated Hospital of USTC approved the present study. The patients/participants provided their written informed consent to participate in this study.

## Author Contributions

Y-zZ: performed trial, data curation and analysis, write original draft. N-aH: designed and performed trial, supervision, edited draft. XY: performed trial, supervision. FJ, M-xL, X-xJ: performed trial and data collection. All authors contributed to the article and approved the submitted version.

## Conflict of Interest

The authors declare that the research was conducted in the absence of any commercial or financial relationships that could be construed as a potential conflict of interest.

## Publisher’s Note

All claims expressed in this article are solely those of the authors and do not necessarily represent those of their affiliated organizations, or those of the publisher, the editors and the reviewers. Any product that may be evaluated in this article, or claim that may be made by its manufacturer, is not guaranteed or endorsed by the publisher.
